# Imperfect Oriented Attachment of Gold Nanoparticles

**DOI:** 10.1002/advs.77335

**Published:** 2026-09-03

**Authors:** Fangjie Meng, Peng Hu, Yongzhao Zhang, Gengshuo Zhang, Jiaqi Zhang, Ronghan Wei, Wen Wang, Shaobo Cheng

**Affiliations:** ^1^ Henan Key Laboratory of Diamond Optoelectronic Materials and Devices, Key Laboratory of Material Physics, School of Physics and Laboratory of Zhongyuan Light, Ministry of Education Zhengzhou University Zhengzhou China; ^2^ School of Mechanics and Safety Engineering Zhengzhou University Zhengzhou China; ^3^ Institute of Quantum Materials and Physics Henan Academy of Sciences Zhengzhou China; ^4^ Department of Material Science Engineering National University of Singapore Singapore Singapore

**Keywords:** coalescence, gold nanoparticle, imperfect oriented attachment, in situ TEM, twin boundary

## Abstract

Particle coalescence is a fundamental pathway of nanoparticle growth, directly governing structural reconstruction, defect formation, and morphological evolution. However, the underlying driving forces behind imperfect oriented attachment, including what initiates the coalescence of misoriented crystals and determines the resulting structures remain unclear, particularly in liquid environments. This study combines in situ liquid‐phase transmission electron microscopy with molecular dynamics calculations to investigate the coalescence dynamics of misoriented gold nanoparticles. Our findings reveal that misoriented nanoparticles can coalesce into a single crystal via a kinetically driven jump‐to‐coalescence process triggered by instantaneous hydration layer collapse. This coalescence is accompanied by a significant potential energy reduction, and the released energy enhances atomic mobility, particularly in small nanoparticles, enabling rapid structural rearrangement into a single crystal. Moreover, the initial contact position relative to the twin boundary determines the atomic diffusion pathway during the coalescence process of twin nanoparticles, while the twin boundary exhibits high stability during the merging process. These findings clarify the regulatory role of the kinetic factor in nanoparticle coalescence behavior and offer guidance for controlling crystal structure and designing twinned nanostructures in the liquid phase synthesis.

## Graphical abstract

The cover illustrates the imperfect oriented attachment of gold nanoparticles in liquid, as revealed by in situ liquid‐phase transmission electron microscopy. Triggered by the abrupt collapse of the interfacial hydration layer, two misoriented nanoparticles undergo a kinetically driven “jump‐to‐coalescence”. They fuse directly into a single crystal without pre‐alignment, revealing a kinetically dominant pathway for nanoparticle coalescence.

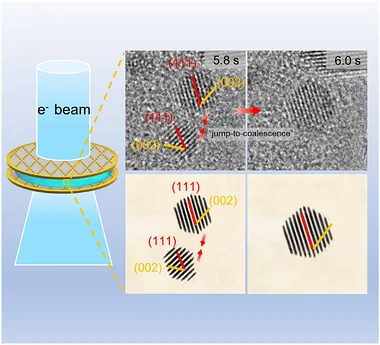

## 1 Introduction

Nanoscience and nanotechnology rely on the precise control over the size, morphology and crystal structure of nanomaterials, as these structural features directly determine their optical, catalytic, electronic, and mechanical properties [1, 2, 3, 4, 5]. Coalescence, and specifically, oriented attachment (OA), is now recognized as a critical pathway for nanoparticle growth, enabling the formation of diverse crystal morphologies including nanorods, chains, and branched nanowires [6, 7, 8]. Nanoparticle coalescence not only determines the size and morphology of the final materials, but also directly affects their crystal integrity, defect structure, and interfacial properties. These factors further govern the stability, catalytic activity, and optical response of the materials [9, 10]. Recent advances in defect engineering and nanoassembly have highlighted that grain boundary formation, defect evolution, and structural reconstruction during coalescence are key to achieving precise control over nanomaterial structure and properties. During OA, particles with complementary facet orientations recognize and rotate to align before joining along a common crystallographic direction, producing single crystals, twins, or larger particles with characteristic defects [11, 12]. The driving forces of OA include the intrinsic factors such as surface free energy reduction [13], dipole‐dipole interactions [14], van der Waals (vdW) forces [15], and electrostatic forces [16], and could be modulated by the external fields, solution conditions (ionic strength, pH, solvent) [17, 18, 19], temperature [20], ligand dynamics [21], particle size and geometry [22, 23].

However, an imperfect oriented attachment has also been documented [24, 25, 26]. In this mechanism, particles attach with a degree of misalignment but can subsequently coalesce into single crystals, multipodal structures, and analogous architectures [27, 28, 29]. Ondry et al. found that imperfect attachment of PbTe nanocrystals on {100}/{110} facets induces edge dislocations and lattice misorientation. Edge dislocations on {100} facets are rapidly eliminated to form defect‐free interfaces, while those on {110} facets persist and transform into mixed dislocations, causing defect retention [28]. Lange et al. showed that for gold nanoparticles (Au NPs) undergoing imperfect OA, the process involves three key stages: the initial formation of quantized grain boundaries with dislocations, followed by low‐barrier dislocation glide that disintegrates these boundaries, ultimately producing nearly single‐crystal aggregates or aggregates with only a few residual twin boundaries [29]. Nelli et al. found that the relative orientation of initial facets is a critical kinetic parameter governing the final structure of gold nanocluster coalescence. At 500 K, when two fcc truncated octahedral (TO) clusters collide with matched (111) facets, the formation probability of decahedral (Dh) structures is only 2%; by contrast, misaligned (111) and (100) facets collisions raise the final Dh formation probability to 24% [30]. To date, fundamental uncertainties persist regarding nanoparticle coalescence. It is still unclear whether coalescence necessarily proceeds via oriented attachment (OA), or if alternative pathways dominate under specific conditions, and the final structure of the fused product often remains unpredictable. The driving force behind imperfect OA remains debated, particularly the roles of thermodynamic versus kinetic mechanisms. Furthermore, the regulatory influence of the hydration layer in liquid environments is poorly understood, and the atomic‐scale diffusion pathways at the contact interface of twin particles during fusion have not been clarified. These uncertainties also limit the ability to design nanomaterials based on coalescence pathways.

Gold nanoparticles are widely used in catalysis, biosensing, and related fields, and their morphology and structure critically determine their performance [31, 32]. In this study, we integrated in situ liquid‐phase transmission electron microscopy (TEM) with molecular dynamics (MD) calculations to investigate the imperfect oriented attachment behavior of gold nanoparticles in water. In situ TEM results showed that nanoparticles can coalesce into single crystals, even though their crystal planes are not well aligned prior to the contact. Quantitative analyses and molecular dynamics calculations revealed the significant role of kinetic factors in this process. Additionally, we further examined how the contact position between twinned gold nanoparticles controls atomic diffusion pathways and the resulting structures. By establishing the correlation among interfacial hydration structure, particle kinetic behavior, and defect evolution, this study proposes a design strategy for defect generation, grain boundary evolution, and structural regulation during the assembly of gold nanoparticles, which provides important guidance for constructing functional nanosystems with excellent catalytic performance.

## 2 Results and Discussion

### 2.1 Experimental Setup of In Situ Liquid Cell TEM

This study employs in situ TEM to systematically investigate the coalescence mechanisms of gold nanoparticles. Initially, gold nanorods with an approximate size of 50 nm were synthesized using the polyol method (Figure S1). Prior to the experiment, the surfactants on the surface of the nanorods were removed using H^+^‐CTA^+^ ion exchange method, followed by their subsequent redispersion in water [33] (see the details in the experimental section). The Au nanorod dispersion was mixed with a 100 mM hydrobromic acid (HBr) aqueous solution at a 1:1 volume ratio. Subsequently, 3 µL of the mixed solution was dropped onto the surface of copper grids coated with an ultrathin amorphous carbon film. Another copper grid was then placed face‐down with its ultrathin carbon film aligned against the first one. Two ultrathin carbon films are bonded and sealed via van der Waals forces to assemble a confined liquid cell. After allowing the liquid cell to rest for 2 to 3 h, a leak test was conducted to verify its sealing integrity. During the experimental procedure, in situ observations of the samples were conducted using a FEI Themis transmission electron microscope (see the details in the experimental section).

Under electron beam irradiation, water undergoes radiolysis, producing oxidizing species such as hydroxyl radicals (OH·), hydroperoxyl radicals (HO_2_·), and hydrogen peroxide (H_2_O_2_), as well as reducing species including solvated electrons (e_h_^−^) and hydrogen radicals (H·) (Note S1) [34]. The oxidizing species can etch gold nanorods, leading to the release of Au ions that complex with bromide ions (Br^−^) [35]. The dose rate under our experimental conditions to approximately ∼10^9^ Gy/s. The etching of gold nanorods leads to local accumulation of dissolved Au species in the thin liquid layer (∼100 nm). Upon reaching supersaturation, gold nanoparticles start to nucleate and precipitate. We observed monomer attachment growth (Figure S2). Nanoparticle coalescence is another widely reported nanoparticle growth pathway [36]. In this work, we focus primarily on growth via coalescence.

### 2.2 The Coalescence between Single Crystals

**FIGURE 1:**
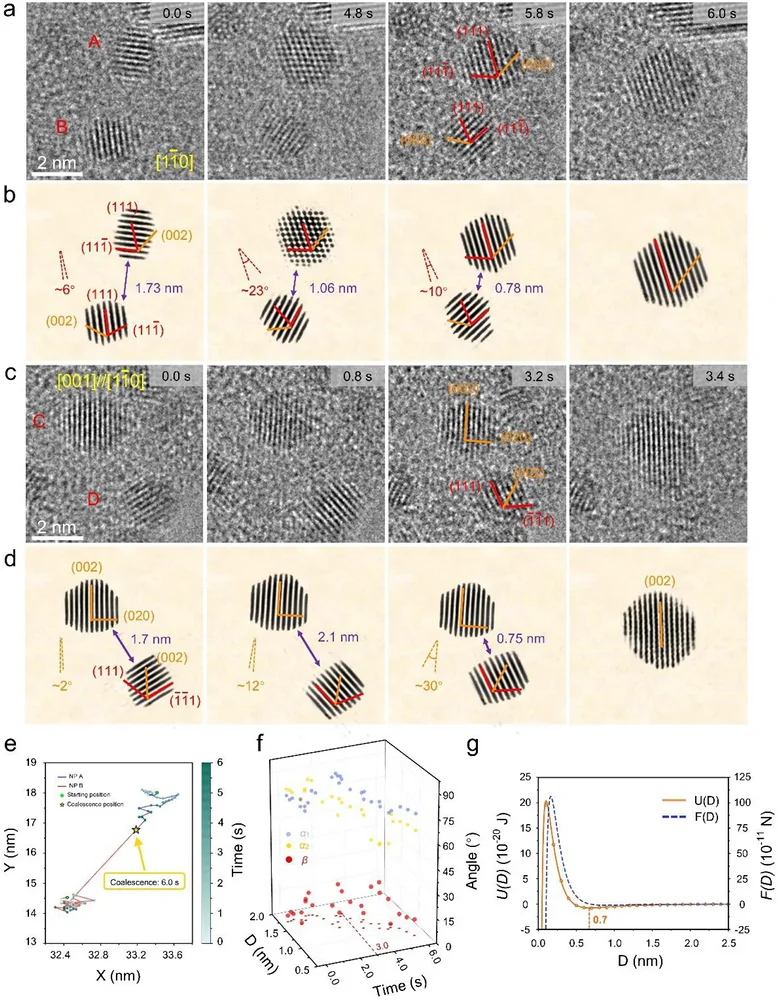
Jump‐to‐coalescence of nanoparticles via imperfect oriented attachment. (a) TEM image series shows the coalescence process between Au NP A and Au NP B. The red and orange lines denote the {111} and {002} facets, respectively. Scale bar, 2 nm. (b) Filtered images corresponding to the TEM images in (a), highlighting the evolution of the orientations and facets of the nanoparticles. (c) Time‐series TEM images of the coalescence process between Au NP C and Au NP D. Scale bar, 2 nm. (d) Filtered images corresponding to the TEM images in (c). (e) Variations of the 2D projected centroid positions of the two gold nanoparticles in Figure 1a. (f) Variations of the angles between the (111) crystal planes of the two particles and the horizontal line (NP A: α_1_, NP B: α_2_), as well as the angle (β) between the (111) crystal planes of the two particles, with time and separation distance for the process in Figure 1a. The red projections in the time‐distance plane denote the separation distance of the two particles at corresponding times. (g) Relationship between the interaction potential (*U*) and interaction force (*F*) of the particle pair with separation distance.

Figure 1a shows the coalescence process between two gold nanoparticles with the $\left[1\bar{1}0\right]$ zone axis (Video S1). Initially, the separation distance between the two particles was 1.73 nm, and the minimum angle between their (111) facets was 6°. Over the subsequent 3 s, the distance between the two nanoparticles fluctuated slightly around 1.5 nm, with no significant change observed. During this period, both nanoparticles exhibited random rotation (Figure 1f). From 3 s, the distance decreased progressively, and reached 0.78 nm at 5.8 s. Then the nanoparticles jumped‐to‐coalesce along the (111) facets to form a new single crystal at 6 s (Figure 1a,e). A notable ∼10° misorientation between the (111) facets was observed at 5.8 s prior to coalescence, confirming imperfect crystallographic alignment (Figure 1b; Figure S3). Furthermore, there is no typical formation of the neck area during this process. This behavior is distinct from previously reported OA pathways, in which nanoparticles either merge into a single crystal via perfect alignment or form a twin boundary or dislocations at the interface when slightly misaligned [37].

In a second scenario, we observed the interaction between particle C, oriented along the [001] zone axis, and particle D, oriented along the $\left[1\bar{1}0\right]$ zone axis (Figure 1c; Video S2). During their approach, the nanoparticles underwent rotation and ultimately jumped to coalesce via their (002) facets at a separation of 0.75 nm. Immediately before coalescence (3.2 s), a significant misorientation angle of 30° was measured between their (002) facets (Figure 1d). This observation deviates from the thermodynamic expectations. While coalescence along the higher‐energy {100} facets is thermodynamically favored for gold nanoparticles (face‐centered cubic structure) due to the greater energy released compared to {111} facets [38]. However, our results demonstrate that coalescence proceeds along both {111} and {100} facets (Figure 1a–d) and can merge into a single crystal without requiring strict facets alignment prior to coalescence. These results imply that thermodynamic factors are not the primary determinants in the coalescence process, and instead, kinetic factors may play a crucial role in governing this phenomenon.

To further investigate the coalescence dynamics, we statistically analyzed the time‐dependent orientations of the (111) facets of the nanoparticles in Figure 1a (Figure 1f). The angles *α*_1_ and *α*_2_ (between the (111) directions of particle A, B and a horizontal axis) and their relative angle *β* all exhibited random fluctuations throughout the observation period. During the initial stable period (0‐3 s) and even as the nanoparticles began to approach (after 3 s), the angle β continued to vary unpredictably. The absence of consistent rotational alignment immediately before coalescence provides direct evidence for the dominance of kinetic processes over thermodynamic alignment.

The jump‐to‐coalescence behavior and its kinetic driving force were further elucidated by analyzing ten similar coalescence events. The mean distance between the nanoparticles before coalescence was measured as 0.64 ± 0.20 nm (Figure S4). This distance is comparable to the separation observed immediately prior to the jump‐to‐contact event during oriented attachment of ferrihydrite nanoparticles under solution conditions, as reported by Li et al. [12]. This sudden jump‐to‐contact event is attributed to the removal of the hydration layer on the particle surface [39]. In a solvent medium, interparticle interactions include van der Waals forces, magnetic forces, electrostatic forces, and steric‐hydration forces [38]. In our experimental system, magnetic, electrostatic, and dipole interactions were excluded (Note S2), and ligand influence was negligible (see the details in the experimental section). Thus, the interparticle potential *U*(*D*) is described by Equation (1), comprising a steric‐hydration repulsion term and a van der Waals attraction term [38]:

(1) $$\begin{array}{cc} & U\left(D\right)=W_{0}e^{-\frac{D}{\lambda }}\\ & +\left\{-\frac{A}{6}\left(\frac{2R^{2}}{\left(4R+D\right)D}+\frac{2R^{2}}{{\left(2R+D\right)}^{2}}+\ln\frac{\left(4R+D\right)D}{{\left(2R+D\right)}^{2}}\right)\right\} \end{array}$$

Based on Equation (1) and the statistics distribution results from twenty nanoparticle pairs (Figure S5 and Note S2), we plotted the interaction potential *U*(*D*) and force *F*(*D*) (Figure 1g). The potential minimum at approximately 0.7 nm represents a dynamic equilibrium between repulsive hydration forces and attractive van der Waals forces, with this equilibrium spacing being essentially equivalent to the separation distances observed in the two nanoparticle pairs prior to imperfect oriented attachment in Figure 1. The critical distance for nanoparticle jump‐to‐coalescence is steadily confined within 0.6‐0.8 nm. Given the typical size of water molecules (∼0.27 nm), this spacing corresponds to approximately 2–3 layers of water molecules [40]. At this separation, water molecules remain mobile on the gold nanoparticle surface and continuously exchange with bulk water molecules [41]. As the nanoparticle separation decreases, steric hindrance leads to the eventual displacement of these confined molecules, ultimately leading to the collapse of the hydration layer.

**FIGURE 2:**
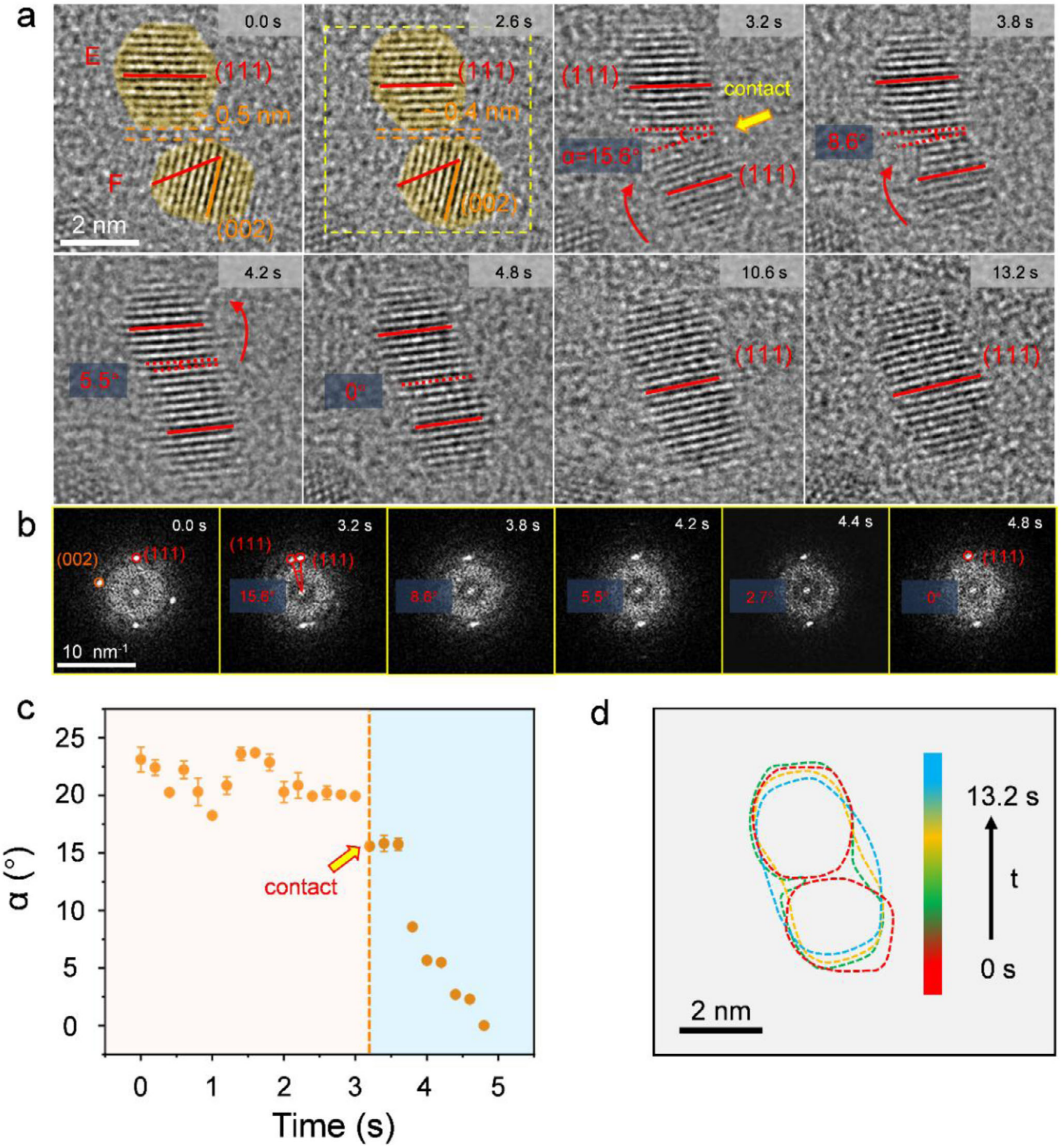
The dynamic process of progressive crystal facet adjustment in oriented attachment. (a) Time‐resolved TEM images show the oriented attachment process of Au NP E and Au NP F. Red and yellow lines indicate the (111) and (002) facets, respectively. The red arrow denotes the rotation direction of the Au NPs. Red dashed lines mark the relative angle between the (111) facets of the two nanoparticles. Scale bar, 2 nm. (b) Fast Fourier Transform (FFT) patterns of the two particles enclosed in the yellow box in (a). Scale bar, 10 nm^−1^. (c) Temporal evolution of the relative angle between the (111) facets of the two particles. (d) Schematic illustration of the shape evolution of the two particles during coalescence from 0 to 13.2 s.

In addition to the jump‐to‐coalescence pathway with misaligned crystal facets, a representative example of the oriented attachment (OA) process was also captured. Figure 2a illustrates that the two nanoparticles form a single crystal through contact, rotation, and lattice alignment (Video S3). Initially (0 s), the interplanar angle between their (111) planes was 23°. Upon forming a contact point at 3.2 s, the angle between the two (111) facets of nanoparticles was measured as 15.6° (Figure 2b). Subsequently, NP F undergoes a clockwise rotation over 0.6 s (until 3.8 s) while NP E remains stationary, reducing the misorientation angle continuously. Upon achieving full contact at 4.0 s, a grain boundary with a 5.6° misorientation persisted. NP F then ceased rotation as NP E began a counterclockwise turn (Figure S6), until their (111) facets reached a coherent orientation with near 0° misalignment by 4.8 s (Figure 2c). Subsequent mass redistribution eliminated the neck region between particles, resulting in ellipsoidal shapes after structural relaxation. To quantify coalescence, we tracked the evolution of the length (l), width (w), and neck diameter (n) of the merged particle (Figure 2d; Figure S7). The findings demonstrate that the coalescence, driven by surface atom diffusion to minimize surface energy [42, 43], governs the morphological evolution during coalescence. Specifically, the radial dimensions (w, n) exhibited a continuous increase at an average rate of 0.036 nm/s, whereas the axial dimension (l) showed a continuous decrease at an average rate of 0.05 nm/s. The filling of the neck region is mainly driven by the collective motion of numerous atoms [30].

It is therefore hypothesized that the coalescence pathways of gold nanoparticles are governed by the interplay between the interfacial energy landscape and hydration layer dynamics. In kinetically dominated jump‐to‐coalescence, nanoparticles have sufficient kinetic energy to rapidly extrude the hydration layer, enabling direct coalescence via van der Waals attraction without the need for crystallographic pre‐alignment. In contrast, when the distance between nanoparticles falls below 0.5 nm, strong compression of the hydration layers raises the system's potential energy above zero (Figure 1g). This indicates that hydration repulsion surpasses van der Waals attraction, while nanoparticle kinetic energy remains inadequate to disrupt the compressed hydration layer. Under such conditions, factors such as the structured interfacial hydration layer, crystal facet‐dependent short‐range interactions, and local anisotropy in interfacial energy collectively induce orientation selective torque, favoring the thermodynamically driven oriented attachment pathway, permitting atomic diffusion and lattice relaxation to proceed. This orientation‐dependent phenomenon is prominent when 0 or 1 layer of confined water layer separates the particles and diminishes rapidly as the number of interstitial water layers increases [44]. Subsequent surface energy minimization drives crystallographic reorientation and neck elimination through surface diffusion. Essentially, the hydration layer imposes a distance‐dependent energy barrier that dictates the coalescence mechanism: at larger separations, higher kinetic energy is required to disrupt the hydration shell and trigger kinetic coalescence; at shorter distances, hydration repulsion is amplified, and limited kinetic energy favors a transition to thermodynamically controlled aligned attachment.

**FIGURE 3:**
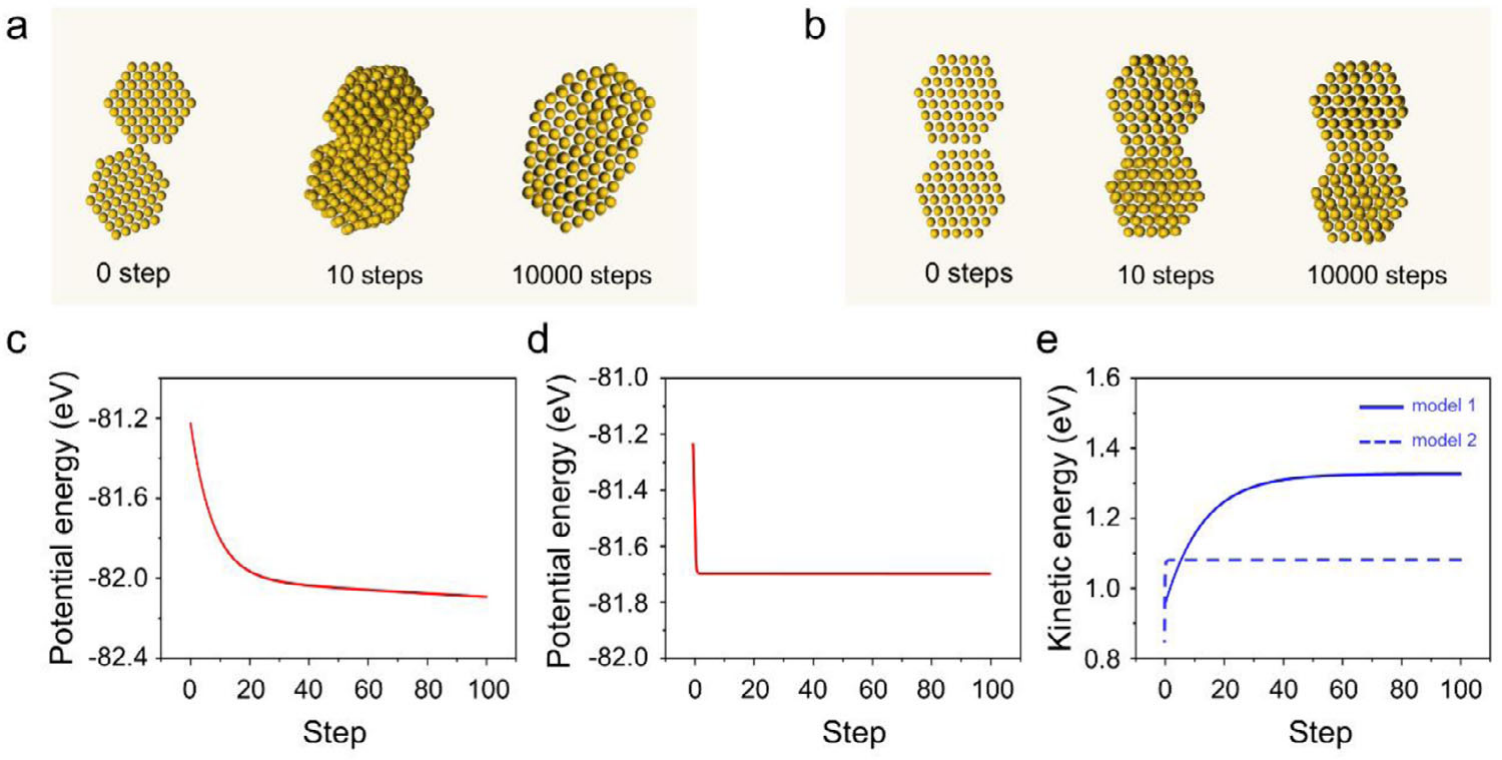
Molecular dynamics calculations of the coalescence process of gold nanoparticles with two initial orientation configurations. (a) Coalescence sequence of particle pairs initially in an angled orientation (model 1). (b) Coalescence sequence of particle pairs initially in a parallel orientation (model 2). (c) Potential energy variation during the coalescence process of particle pairs initially in an angled orientation under the NVT (canonical) ensemble. (d) Potential energy variation during the coalescence process of particle pairs initially in a parallel orientation in the NVT (canonical) ensemble. (e) Kinetic energy variation during the coalescence process of particle pairs with two different initial orientations in the NVE (microcanonical) ensemble.

To elucidate the role of facet alignment in nanoparticle coalescence, we performed molecular dynamics (MD) calculations to monitor the structural and energetic evolution during fusion (Figure 3; Note S3). We systematically compared two distinct initial configurations: (1) the particles were placed with a 60° angle between (111) facets (Figure 3a); (2) particles with parallel (111) facets (Figure 3b). Simulations were carried out in the NVT (canonical) ensemble at 300 K (Figure S8). Results show that the nanoparticles can coalesce into single crystals in both configurations. When the (111) planes are initially misaligned, attractive forces drove the particles to coalesce, inducing local atomic rearrangements and a transient disordered state before they ultimately fuse into a single crystal. By contrast, when the (111) planes are parallel, the nanoparticles coalesce rapidly, and the structural order is preserved throughout the process without significant disordering (Figure 3b). Correspondingly, in the misaligned case the potential energy decreases gradually and then stabilizes as the system passes through a transient disordered state and subsequently reorders (Figure 3c), whereas in the parallel case the energy drops sharply and quickly reaches a stable value, indicating a kinetically favorable fusion that preserves structural order (Figure 3d).

The coalescence of misoriented nanoparticles leads to a significantly greater reduction in potential energy (0.97 eV) than that of oriented ones (0.44 eV). This substantial energy release primarily originates from the annihilation of the high‐energy mismatched interface (Figure 3c,d). Our additional NVE (microcanonical) ensemble simulations (Figure S9) further revealed a pronounced energy exchange: a decrease in potential energy (Δ*E_p_* = −0.4 eV) was coupled with an increase in kinetic energy (Δ*E_k_* = +0.4 eV). This conversion of interfacial mismatch energy into localized kinetic energy serves as a potent thermodynamic driving force, which significantly enhances the mobility of interfacial atoms, thereby promoting surface diffusion and facilitating the rapid structural rearrangement into a single crystal. To elucidate the role of trace residual Br^−^, two hydronium (H_3_O^+^) and two Br^−^ ions were introduced into the close‐contact interface between the two Au nanoparticles for both model 1 and model 2 (Figure S10 and Note S3). Under this condition, initially misoriented nanoparticles still undergo complete coalescence into single crystals, driven by substantial energy release. It is noteworthy that the formation of single crystals through rapid surface diffusion during the coalescence of misaligned nanoparticles occurs exclusively in the small particles. In larger nanoparticles (diameters > 5 nm), the significantly reduced surface‐to‐volume ratio diminishes the dominance of surface diffusion, hindering the elimination of interfacial misfit and typically leading to the generation of stacking faults and dislocations at the boundary (Figure S11).

### 2.3 The Coalescence of Twinned Nanoparticles

**FIGURE 4:**
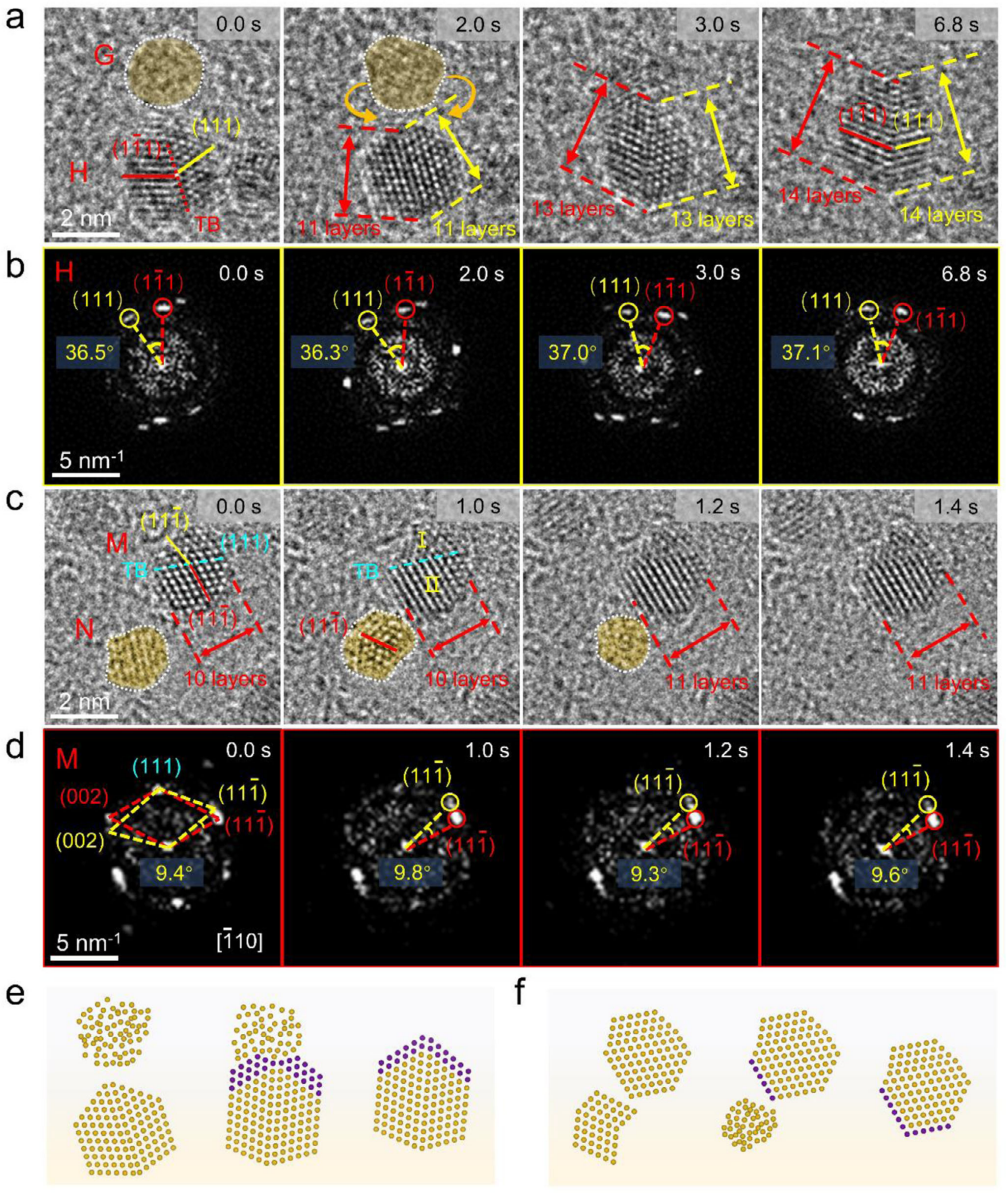
The coalescence process between twinned gold nanoparticles. (a) Time‐resolved TEM images illustrating the coalescence between the nanoparticle G and the twinned nanoparticle H. The red and yellow lines indicate the $\left(1\bar{1}1\right)$ and (111) facets, respectively. Red and yellow arrows indicate the number of atomic layers on either side of the twin boundary in particle H. Scale bar, 2 nm. (b) FFT image of the twinned particle H depicted in Figure 4a. Scale bar, 5 nm^−^^1^. (c) Time‐resolved TEM images illustrating the coalescence process between twinned nanoparticle M and twinned nanoparticle N. The red arrow indicates the number of atomic layers in domain II of twinned particle M along the direction perpendicular to the $\left(11\bar{1}\right)$ plane. The yellow shaded region represents the portion of twinned particle N that has not yet fully diffused. Scale bar, 2 nm. (d) FFT image of the twinned particle M depicted in Figure 4c. Scale bar, 5 nm^−1^. (e,f) Schematic diagrams of different coalescence modes for the two groups of particles (Figure 4a,c). The purple spheres represent the newly added atoms.

Besides, the coalescence process of twinned nanoparticles was also investigated (Figure 4). Figure 4a presents the dynamic coalescence between nanoparticle G and the twinned nanoparticle H (Video S4). At 0 s, the facets on both sides of twinned nanoparticle H were (111), and a projected interplanar angle of 36.5° was measured, as shown in Figure 4b. The distance between the particles decreased to ∼0.4 nm by 2.4 s, and the two particles made contact at 3.0 s. During the coalescence process, twinned nanoparticle H preserved its twin structure, and particle G made contact with the twin boundary of particle H. The merged particle developed an upper region of high disorder and a lower region that retained a well‐defined twin structure. The atomic layers on both sides of the twin increased to 13 with a {111} plane angle of 37°. These results indicate that when nanoparticle G comes into contact with the twin boundary (TB) of twinned nanoparticle H, the atoms of nanoparticle G diffused into the two twin domains of nanoparticle H, and the contact interface of the two particles underwent reconstruction, thereby driving the upward migration of the coalescence front. By 6.8 s, particle G had fully diffused into particle H, and the fused particle preserved the twin structure with a projected {111} angle of 37.1°. The minimal change in the {111} angle throughout coalescence suggests that the twin configuration remained stable and that the released energy was insufficient to alter it.

It has been reported that the initial contact modes of five‐fold twins regulate their coalescence pathways and final configurations [45]. We therefore examined coalescence between two other twinned nanoparticles (Figure 4c), where the smaller nanoparticle N was positioned adjacent to domain II of twinned nanoparticle M and far from its twin boundary. Two nanoparticles gradually approached each other and formed a contact point at 1.0 s. During the coalescence process, atoms from nanoparticle N diffused into nanoparticle M, ultimately leading to an increase in the number of atomic layers in domain II of twinned nanoparticle M from 10 to 11 layers (Figure 4c,f), while the projected area of domain I showed no measurable change (Figure S12). These results indicate that when twinned nanoparticle N is far from the twin boundary of twinned nanoparticle M, its atoms only diffuse into the adjacent domain II of twinned nanoparticle M, and do not diffuse into twin domain I. Additionally, the projected angle between the {111} planes on both sides of twinned M remained essentially constant, suggesting that its crystal structure remained stable without significant alteration.

The initial contact position between twinned nanoparticles plays a decisive role in their diffusion behavior within the twin structure. When a particle directly contacts the twin boundary of the twinned nanoparticle (Figure 4a), its atoms diffuse simultaneously into the twin domains on both sides, whereas when the contact point of the particle was far from the twin boundary (Figure 4c), its atoms tend to incorporate into the adjacent single twin domain without crossing the twin boundary. This difference in diffusion paths, which depends on the initial contact position, profoundly reveals that the twin boundary plays a key role in directional selection during atomic migration. More importantly, regardless of the contact mode, both sets of relatively large twinned crystals maintain twin angle stability, indicating that the symmetrical orientation relationship of twins can efficiently dissipate coalescence energy.

## 3 Conclusions

Coalescence pathways, and in particular imperfect oriented attachment of gold nanoparticles, were investigated by combining in situ liquid‑phase TEM with MD calculations. Experimental results reveal that though crystal facets are misaligned, the nanoparticles can undergo an essentially instantaneous jump‐to‐coalescence process that yields a single crystal. This pathway is kinetically driven, with random rotations and transient kinetic energy disrupting the hydration shell and allowing van der Waals attraction to induce rapid fusion. In contrast, classical oriented attachment is thermodynamically controlled and proceeds via gradual atomic diffusion and lattice relaxation to reach a stable single crystal state. MD calculations show that coalescence of nanoparticles with misaligned (111) facets traverses brief disordered intermediates in which potential energy converts to kinetic energy, enhancing the mobility of surface atoms and driving recrystallization. For twinned nanoparticles, the contact position dictates, which twin domain incorporates atoms while the twin boundary remains stable and helps dissipate coalescence energy.

## 4 Experimental Section

### 4.1 TEM Characterization

The TEM Characterization and in situ experiments were carried out using FEI Themis with a Cs‐corrector operated at 300 kV. The primary objective of this in situ study is to observe the coalescence and growth of gold nanoparticles after their formation. In this context, the electron beam primarily serves as an imaging source to track the dynamic evolution of particles. The incident electron flux density during the observation of particle coalescence is 1000–1600 e^−^·Å^−2^·s^−1^. This corresponds to an estimated electron beam dose rate of 4.8 × 10^9^–7.6 × 10^9^ Gy/s (see Note S4 for calculation).

### 4.2 Removal of Surface Ligands

Gold nanorods were synthesized via the polyol method and were subsequently washed three times with deionized water to eliminate excess surface surfactant, cetyltrimethylammonium bromide (CTAB). The CTAB molecules on the surface of the gold nanorods were removed using an H^+^‐CTA^+^ ion exchange approach. Initially, the gold nanorod solution was mixed with 50 mM hydrobromic acid at a volume ratio of 1:1 and stirred under an argon atmosphere at room temperature for 5 min. Subsequently, the mixture was centrifuged at 10 000 rpm for 10 min to remove the supernatant, and the gold nanorods were redispersed in deionized water.

### 4.3 Theoretical Study

This study employed molecular dynamics calculations to investigate the coalescence process of two truncated octahedral gold nanoparticles, each with a diameter of 2.0 nm and a total of 260 atoms. Two initial configurations were constructed: in Model 1, the minimum center‐to‐center distance between atoms of the two particles is 0.3 nm, with the (111) crystal facets oriented at an angle; in Model 2, the interparticle spacing is 0.5 nm, and the (111) facets are parallel. Simulations were conducted under both NVT ensemble (constant number of particles, volume, and temperature) and NVE ensemble (constant number of particles, volume, and energy), with an initial temperature set at 300 K. Based on Au nanoparticles with a diameter of 2.0 nm, ionic species were incorporated to construct ion‐containing simulation systems, wherein two H_3_O^+^ and two Br^−^ ions were inserted at the intimate contact interface of the two nanoparticles, and rigid‐body constraints were imposed on all H_3_O^+^ throughout simulations. In Model 1, the nanoparticles were initially placed 0.7 nm apart (closest atom centers), while Model 2 adopted an initial separation of 0.9 nm. Simulations were performed at 300 K under the NVT ensemble, with multiple combined force fields adopted to describe atomic interactions. Specifically, interionic interactions were characterized by the Madrid‐2019 force field [46, 47], whereas Au‐ion cross‐interactions were defined via Lennard‐Jones and Coulombic potentials. The relevant potential parameters were derived from the CVFF‐interface force field and converted using the arithmetic mixing rules [48].

Further, we performed another set of molecular dynamics fusion simulations of two truncated octahedral gold nanoparticles: each with a diameter of 5.9 nm (containing 4957 atoms), with a certain angle of misorientation between their (111) planes, a closest atom centers distance of 0.3 nm, and the simulations were conducted at 900 K in the NVT ensemble. Atomic interactions were described using the embedded‐atom method (EAM) potential developed by Foiles et al. [49], and a time step of 1 ps was employed. All simulations were performed using the LAMMPS software package [50].

## Author Contributions

**Wen Wang** and **Shaobo Cheng** conceived and designed the experiments. **Wen Wang** performed the experiments; **Fangjie Meng** developed the simulations. **Yongzhao Zhang**, **Gengshuo Zhang**, and **Jiaqi Zhang** took part in the discussion and data analysis; **Fangjie Meng** and **Wen Wang** wrote paper with the contributions of all authors. **Shaobo Cheng** revised the paper. All authors have given approval to the final version of the manuscript.

## Conflicts of Interest

The authors declare no conflicts of interest.

## Supporting information




**Supporting File 1**: advs77335‐sup‐0001‐SuppMat.docx.

**Supporting File 2**: advs77335‐sup‐0002‐SuppMat.docx.

**Supporting File 3**: advs77335‐sup‐0003‐S1.mp4.

**Supporting File 4**: advs77335‐sup‐0004‐S2.mp4.

**Supporting File 5**: advs77335‐sup‐0005‐S3.mp4.

**Supporting File 6**: advs77335‐sup‐0006‐S4.mp4.

## Data Availability

The data that support the findings of this study are available from the corresponding author upon reasonable request.

## References

[1] F. Tao and P. A. Crozier , “Atomic‐Scale Observations of Catalyst Structures under Reaction Conditions and during Catalysis,” Chemical Reviews 116, no. 6 (2016): 3487–3539, 10.1021/cr5002657. 26955850

[2] R. W. Song , T. C. Jiang , X. Y. Zhang , C. L. Shen , Q. Lou , and C. X. Shan , “Triplet Electron Exchange in Carbon Nanodots‐Assisted Long‐Persistent near‐Infrared Chemiluminescence for Oncology Synergistic Imaging and Therapy,” Advanced Science 12, no. 5 (2025): 2411898, 10.1002/advs.202411898. 39661728 PMC11791938

[3] Y. D. Qu , Y. Zhang , P. Ni , C. Shan , D. Hunger , and K. Mølmer , “Superradiance From Nitrogen‐vacancy Centers Coupled to an Ultranarrow Optical Cavity,” Physical Review A 111, no. 3 (2025): 033711, 10.1103/PhysRevA.111.033711.

[4] C. Liu , K. Hongo , R. Maezono , J. Zhang , and Y. Oshima , “Stiffer Bonding of Armchair Edge in Single‐Layer Molybdenum Disulfide Nanoribbons,” Advanced Science 10, no. 30 (2023): 2303477, 10.1002/advs.202303477. 37697633 PMC10602518

[5] J. Li , P. Hu , F. Meng , J. Tang , and W. Wang , “Nonclassical Nucleation of Silver Nanoparticles,” Journal of Physics D: Applied Physics 59, no. 19 (2026): 195302, 10.1088/1361-6463/ae651a.

[6] M. Dong , W. Wang , W. Wei , et al., “Understanding the Ensemble of Growth Behaviors of Sub‐10‐nm Silver Nanorods Using in Situ Liquid Cell Transmission Electron Microscopy,” The Journal of Physical Chemistry C 123, no. 34 (2019): 21257–21264, 10.1021/acs.jpcc.9b05267.

[7] H. G. Liao , L. Cui , S. Whitelam , and H. Zheng , “Real‐time Imaging of Pt_3_Fe Nanorod Growth in Solution,” Science 336, no. 6084 (2012): 1011–1014, 10.1126/science.1219185. 22628649

[8] P. Sun , X. Zhang , C. Wang , Y. Wei , L. Wang , and Y. Liu , “Rutile TiO_2_ Nanowire Array Infiltrated with Anatase Nanoparticles as Photoanode for Dye‐sensitized Solar Cells: Enhanced Cell Performance via the Rutile–anatase Heterojunction,” Journal of Materials Chemistry A 1, no. 10 (2013): 3309–3314, 10.1039/c2ta01432g.

[9] L. Wang , L. Wang , X. Meng , and F.‐S. Xiao , “New Strategies for the Preparation of Sinter‐Resistant Metal‐Nanoparticle‐Based Catalysts,” Advanced Materials 31, no. 50 (2019): 1901905, 10.1002/adma.201901905. 31478282

[10] P. Grammatikopoulos , “Progress in Atomistic Modelling of Nanoparticle Coalescence,” Advances in Physics: X 11 (2026): 2611995.

[11] S. Y. Hou , C. Y. Huang , S. B. Tsai , J. Y. Chen , and W. W. Wu , “In Situ Atomic‐scale TEM Observation of Ag Nanoparticle‐mediated Coalescence in Liquids,” Applied Surface Science 546 (2021): 149057.

[12] D. Li , M. H. Nielsen , J. R. I. Lee , C. Frandsen , J. F. Banfield , and J. De Yoreo J , “Direction‐specific Interactions Control Crystal Growth by Oriented Attachment,” Science 336, no. 6084 (2012): 1014–1018, 10.1126/science.1219643. 22628650

[13] W. Lv , W. He , X. Wang , et al., “Understanding the Oriented‐attachment Growth of Nanocrystals From an Energy Point of View: a Review,” Nanoscale 6, no. 5 (2014): 2531–2547, 10.1039/C3NR04717B. 24481078

[14] J. S. Chen , T. Zhu , C. M. Li , and X. W. Lou , “Building Hematite Nanostructures by Oriented Attachment,” Angewandte Chemie International Edition 50, no. 3 (2011): 650–653, 10.1002/anie.201005365. 21226144

[15] A. Ghezelbash , B. Koo , and B. A. Korgel , “Self‐assembled Stripe Patterns of CdS Nanorods,” Nano Letters 6, no. 8 (2006): 1832–1836, 10.1021/nl061035l. 16895382

[16] H. Zhang and J. F. Banfield , “Interatomic Coulombic Interactions as the Driving Force for Oriented Attachment,” CrystEngComm 16, no. 8 (2014): 1568–1578, 10.1039/C3CE41929K.

[17] L. Liu , E. Nakouzi , M. L. Sushko , et al., “Connecting Energetics to Dynamics in Particle Growth by Oriented Attachment Using Real‐time Observations,” Nature Communications 11, no. 1 (2020): 1045, 10.1038/s41467-020-14719-w. PMC7042275 32098968

[18] J. Lee , E. Nakouzi , D. Xiao , et al., “Interplay between Short‐ and Long‐Ranged Forces Leading to the Formation of Ag Nanoparticle Superlattice,” Small 15, no. 33 (2019): 1901966, 10.1002/smll.201901966. 31225719

[19] X. Li , W. X. Chen , J. Zhao , W. Xing , and Z. D. Xu , “Microwave Polyol Synthesis of Pt/CNTs Catalysts: Effects of pH on Particle Size and Electrocatalytic Activity for Methanol Electrooxidization,” Carbon 43, no. 10 (2005): 2168–2174, 10.1016/j.carbon.2005.03.030.

[20] F. Cheng , L. Lian , L. Li , et al., “Hybrid Growth Modes of PbSe Nanocrystals with Oriented Attachment and Grain Boundary Migration,” Advanced Science 6, no. 9 (2019): 1802202, 10.1002/advs.201802202. 31065525 PMC6498134

[21] Y. Bae , K. Lim , S. Kim , et al., “Ligand‐dependent Coalescence Behaviors of Gold Nanoparticles Studied by Multichamber Graphene Liquid Cell Transmission Electron Microscopy,” Nano Letters 20, no. 12 (2020): 8704–8710, 10.1021/acs.nanolett.0c03517. 33186041

[22] P. F. Damasceno , M. Engel , and S. C. Glotzer , “Predictive Self‐assembly of Polyhedra into Complex Structures,” Science 337, no. 6093 (2012): 453–457, 10.1126/science.1220869. 22837525

[23] H. Sun , R. Jiao , H. Xu , G. An , and D. Wang , “The Influence of Particle Size and Concentration Combined with pH on Coagulation Mechanisms,” Journal of Environmental Sciences 82 (2019): 39–46, 10.1016/j.jes.2019.02.021. 31133268

[24] J. De Yoreo J , P. U. P. A. Gilbert , N. A. J. M. Sommerdijk , et al., “Crystallization by Particle Attachment in Synthetic, Biogenic, and Geologic Environments,” Science 349 (2015): aaa6760. 26228157 10.1126/science.aaa6760

[25] J. Li , J. Chen , H. Wang , et al., “In Situ Atomic‐Scale Study of Particle‐Mediated Nucleation and Growth in Amorphous Bismuth to Nanocrystal Phase Transformation,” Advanced Science 5, no. 6 (2018): 1700992, 10.1002/advs.201700992. 29938178 PMC6010897

[26] R. L. Penn and J. F. Banfield , “Imperfect Oriented Attachment: Dislocation Generation in Defect‐free Nanocrystals,” Science 281, no. 5379 (1998): 969–971, 10.1126/science.281.5379.969. 9703506

[27] P. Ren , Z. Lu , M. Song , et al., “Formation of Pyrophosphates across Grain Boundaries Induces the Formation of Mismatched but Oriented Interfaces in Silver Phosphate Polypods,” Applied Surface Science 563 (2021): 149980, 10.1016/j.apsusc.2021.149980.

[28] J. C. Ondry , M. R. Hauwiller , and A. P. Alivisatos , “Dynamics and Removal Pathway of Edge Dislocations in Imperfectly Attached PbTe Nanocrystal Pairs: toward Design Rules for Oriented Attachment,” ACS Nano 12, no. 4 (2018): 3178–3189, 10.1021/acsnano.8b00638. 29470056

[29] A. P. Lange , A. Samanta , T. Y. Olson , and S. Elhadj , “Quantized Grain Boundary States Promote Nanoparticle Alignment during Imperfect Oriented Attachment,” Small 16, no. 29 (2020): 2001423, 10.1002/smll.202001423. 32519454

[30] D. Nelli , G. Rossi , Z. Wang , R. E. Palmer , and R. Ferrando , “Structure and Orientation Effects in the Coalescence of Au Clusters,” Nanoscale 12, no. 14 (2020): 7688–7699, 10.1039/C9NR10163B. 32211622

[31] P. Guo and Y. Gao , “Coalescence of Au Nanoparticles without Ligand Detachment,” Physical Review Letters 124, no. 6 (2020): 066101, 10.1103/PhysRevLett.124.066101. 32109082

[32] H. Qian , M. Zhu , Z. Wu , and R. Jin , “Quantum Sized Gold Nanoclusters with Atomic Precision,” Accounts of Chemical Research 45, no. 9 (2012): 1470–1479, 10.1021/ar200331z. 22720781

[33] W. Wang , D. Song , F. Meng , et al., “Facet‐Dependent Cold Welding of Au Nanorods Revealed by Liquid Cell Transmission Electron Microscopy,” Advanced Science 12, no. 12 (2025): 2412779, 10.1002/advs.202412779. 39888289 PMC11948076

[34] B. Fritsch , S. Lee , A. Körner , N. M. Schneider , F. M. Ross , and A. Hutzler , “The Influence of Ionizing Radiation on Quantification for in Situ and Operando Liquid‐Phase Electron Microscopy,” Advanced Materials 37, no. 13 (2025): 2415728, 10.1002/adma.202415728. 39981755 PMC11962711

[35] W. Wang , T. Xu , J. Chen , et al., “Solid–liquid–gas Reaction Accelerated by Gas Molecule Tunnelling‐Like Effect,” Nature Materials 21, no. 8 (2022): 859–863, 10.1038/s41563-022-01261-x. 35618827

[36] H. Zheng , R. K. Smith , Y. W. Jun , C. Kisielowski , U. Dahmen , and A. P. Alivisatos , “Observation of Single Colloidal Platinum Nanocrystal Growth Trajectories,” Science 324, no. 5932 (2009): 1309–1312, 10.1126/science.1172104. 19498166

[37] D. Li , Q. Chen , J. Chun , K. Fichthorn , J. De Yoreo , and H. Zheng , “Nanoparticle Assembly and Oriented Attachment: Correlating Controlling Factors to the Resulting Structures,” Chemical Reviews 123 (2023): 3127–3159. 36802554 10.1021/acs.chemrev.2c00700

[38] C. Zhu , S. Liang , E. Song , et al., “In‐situ Liquid Cell Transmission Electron Microscopy Investigation on Oriented Attachment of Gold Nanoparticles,” Nature Communications 9 (2018): 421. 10.1038/s41467-018-02925-6 PMC5788991 29379109

[39] U. Anand , J. Lu , D. Loh , Z. Aabdin , and U. Mirsaidov , “Hydration Layer‐Mediated Pairwise Interaction of Nanoparticles,” Nano Letters 16, no. 1 (2016): 786–790, 10.1021/acs.nanolett.5b04808. 26709603

[40] E. Nakouzi , A. G. Stack , S. Kerisit , et al., “Moving beyond the Solvent‐tip Approximation to Determine Site‐specific Variations of Interfacial Water Structure through 3D Force Microscopy,” The Journal of Physical Chemistry C 125, no. 2 (2021): 1282–1291, 10.1021/acs.jpcc.0c07901.

[41] D. T. Limmer , A. P. Willard , P. A. Madden , and D. Chandler , “Water Exchange at a Hydrated Platinum Electrode Is Rare and Collective,” The Journal of Physical Chemistry C 119, no. 42 (2015): 24016–24024, 10.1021/acs.jpcc.5b08137.

[42] D. Nelli , M. Cerbelaud , R. Ferrando , and C. Minnai , “Tuning the Coalescence Degree in the Growth of Pt–Pd Nanoalloys,” Nanoscale Advances 3, no. 3 (2021): 836–846, 10.1039/D0NA00891E. 36133833 PMC9416879

[43] Y. Shen , S. Su , T. Xu , K. Yin , K. Yang , and L. Sun , “In Situ Transmission Electron Microscopy Investigation on Oriented Attachment of Nanodiamonds,” Nano Letters 23 (2023): 9602–9608. 37812081 10.1021/acs.nanolett.3c03300

[44] S. N. Kerisit and J. De Yoreo J , “Effect of Hydrophilicity and Interfacial Water Structure on Particle Attachment,” The Journal of Physical Chemistry C 124, no. 9 (2020): 5480–5488, 10.1021/acs.jpcc.9b12053.

[45] H. Wang , M. Mutailipu , L. Boddapati , A. Samanta , F. L. Deepak , and J. Li , “Atomic‐Scale Dynamics of Five‐Fold Twin Mediated Coalescence: Pathway‐Dependent and Defect‐Governed Nonclassical Growth Mechanisms,” Journal of the American Chemical Society 147, no. 25 (2025): 22104–22114, 10.1021/jacs.5c06375. 40490868

[46] S. Blazquez , M. de Lucas , C. Vega , and F. Gámez , “Acidifying the Madrid‐2019 Force Field: a Rigid Model for H_3_O^+^ with Scaled Charges,” The Journal of Chemical Physics 162, no. 17 (2025): 171101, 10.1063/5.0267223. 40326599

[47] S. Blazquez , M. M. Conde , J. L. F. Abascal , and C. Vega , “The Madrid‐2019 Force Field for Electrolytes in Water Using TIP4P/2005 and Scaled Charges: Extension to the Ions F^−^, Br^−^, I^−^, Rb^+^, and Cs^+^ ,” The Journal of Chemical Physics 156 (2022): 044505. 35105066 10.1063/5.0077716

[48] H. Heinz , T.‐J. Lin , R. Kishore Mishra , and F. S. Emami , “Thermodynamically Consistent Force Fields for the Assembly of Inorganic, Organic, and Biological Nanostructures: the INTERFACE Force Field,” Langmuir 29, no. 6 (2013): 1754–1765, 10.1021/la3038846. 23276161

[49] S. M. Foiles , M. I. Baskes , and M. S. Daw , “Embedded‐atom‐method Functions for the Fcc Metals Cu, Ag, Au, Ni, Pd, Pt, and Their Alloys,” Physical Review B 33, no. 12 (1986): 7983–7991, 10.1103/PhysRevB.33.7983. 9938188

[50] S. Plimpton , “Fast Parallel Algorithms for Short‐Range Molecular Dynamics,” Journal of Computational Physics 117, no. 1 (1995): 1–19, 10.1006/jcph.1995.1039.

