# Supplementary material for: Imperfect Oriented Attachment of Gold Nanoparticles
Source: Adv Sci (Weinh). 2026 Sep 3:e77335. Online ahead of print. doi: 10.1002/advs.77335 (PMC13542399; doi:10.1002/advs.77335)
Supplement: Supplementary file 2 [file ADVS-9999-e77335-s004.docx]

**Supporting Information for**

**Imperfect oriented attachment of gold nanoparticles**

*Fangjie Meng**^1,2^, Peng Hu^1,2^, Yongzhao Zhang^3^, Gengshuo Zhang^4^, Jiaqi Zhang^1^, Ronghan Wei^2^, Wen Wang^1,2*^, Shaobo Cheng^1,3*^*

^1^ Henan Key Laboratory of Diamond Optoelectronic Materials and Devices, Key Laboratory of Material Physics, Ministry of Education, School of Physics and Laboratory of Zhongyuan Light, Zhengzhou University, Zhengzhou 450052, China

^2^ School of Mechanics and Safety Engineering, Zhengzhou University, Zhengzhou 450001, China

^3^ Institute of Quantum Materials and Physics, Henan Academy of Sciences, Zhengzhou, 450046, China

^4^ Department of Material Science Engineering, National University of Singapore, Singapore, Singapore

*Correspondence to: wangw@zzu.edu.cn; chengshaobo@zzu.edu.cn

**Contents**

**Section 1**

Supplementary Videos Captions S1 to S4

**Section 2**

Supplementary Notes 1 to 4

**Section 3**

Supplementary Figures and Figure Captions S1 to S12

**References**

**Section 1**

**Captions for Videos**

**Video S1:** Two gold nanoparticles (NPs) with a ~10° pre-coalescence (111) facet misorientation undergo jump-to-coalescence along (111).

**Video S2:** Two Au NPs with a ~30° pre-coalescence (002) facet misorientation undergo jump-to-coalescence along (002).

**Video S3:** The coalescence process of two Au NPs rotating and aligning along the (111) facet.

**Video S4:** The coalescence process of Au NP G contacting the twin boundary of twinned Au NP H.

**Notes:** All supplementary videos have a playback speed of 1.3 times the actual experimental observation speed, with their presented time scale compressed by this ratio. The key experimental phenomena are consistent with the actual observations.

**Section 2**

**Supplementary Note 1: Effects of Electron Beam**

In situ transmission electron microscopy serves as a pivotal visualization technique for real-time observation of the dynamic evolution of the nanoparticles, achieving imaging with high spatiotemporal resolution through the interaction between high-energy electron beams and matter. On this basis, liquid-phase transmission electron microscopy (LP-TEM) has been developed. By encapsulating liquid samples within a liquid cell, LP-TEM enables the real-time observation of the dynamic evolution of nanomaterials in a liquid environment. However, the high-energy electron beam simultaneously induces three types of effects: solvent radiolysis, localized heating, and atomic collisions. These effects perturb the intrinsic reaction environment of the system from the perspectives of chemical conditions, thermodynamic state, and material structure.

When an electron beam passes through the water layer, it randomly excites water molecules along its trajectory. The average distance between these excitations equals the inelastic mean free path of electrons in water. The excited products immediately react and diffuse, forming spherically expanding high-energy regions called spurs [1]. The evolution within each spur proceeds in three stages: the physical stage (1 fs), where inelastic scattering induces ionization and excitation of water molecules; the physicochemical stage (1 ps), where active species relax to form inhomogeneous primary radiolysis products; and the chemical stage, where the products diffuse and react to ultimately yield steady-state radiolysis products [2, 3].The primary products of electron beam-induced water radiolysis are categorized into radical and non-radical species. Radical species include the strongly oxidizing OH·, weakly oxidizing HO_2_·, and strongly reducing e_h_^-^ and H·. Non-radical species include H_2_O_2_, H_2_, H^+^, and OH^−^. [4]. In an HBr aqueous solution system, bromide ions (Br^−^), acting as radical scavengers, rapidly react with hydroxyl radicals generated by water radiolysis, producing secondary oxidative species such as bromine radicals (Br⋅) and dibromide radical anions (Br_2_⋅^−^). These species are the dominant agents responsible for the etching of gold nanorods. On the other hand, strongly reducing species, including (e_h_^−^) and ·H, can effectively reduce Au^3+^/Au^+^ in solution, thereby initiating the nucleation and growth of gold.

A 300 kV electron beam can transfer a maximum energy of only 4.3 eV to the gold atoms through elastic collisions, which is substantially lower than the displacement threshold energy of gold atoms [5, 6]. Consequently, this energy transfer is insufficient to induce atomic displacements or lattice damage. Second, theoretical estimates for the liquid cell yield a temperature rise of only a few degrees Celsius, which is negligible [1, 4, 7].

**Supplementary Note 2: Interactions between Nanoparticles**

Within the framework of this study, the interparticle forces potentially present including magnetic forces, electrostatic forces, van der Waals attraction, and hydration layer repulsion [8, 9]. Based on the findings of Zhu et al. [10], we posit that neither magnetic nor electrostatic forces serve as the primary interactions in this system. Given that gold nanoparticles are non-magnetic materials, magnetic forces can be considered negligible. Electrostatic forces are also excluded because the precipitated particles exhibit highly symmetrical shapes (truncated octahedra) and lack ligand adsorption, resulting in surface charges that do not generate effective dipole moments. Furthermore, the absence of exogenous electrolytes in the pure water environment leads to a very low surface charge density on the particles. According to the Derjaguin – Landau − Verwey − Overbeek (DLVO) theory [11], this low charge density corresponds to an interaction range that is significantly shorter than the typical interparticle distances observed in the system, rendering the electrostatic force insufficient to dominate particle behavior. Consequently, the interparticle forces in this system are limited to van der Waals attraction and hydration repulsion.

The strength of the interaction forces between particles can be determined from the distribution of the distances between particles, using the Boltzmann distribution. The expression is as follows [12]:

$P(D)=C\exp\left(\frac{-U(D)}{k_{B}T}\right)$ (1)

The relationship between the separation distance of particle pairs and their corresponding distribution is [10]:

$P(D)=C\exp\left\{-\frac{W_{0}}{k_{B}T}e^{-\frac{D}{\lambda}}+\frac{A}{6k_{B}T}\left(\frac{2R^{2}}{(4R+D)D}+\frac{2R^{2}}{(2R+D)^{2}}+\ln\frac{(4R+D)D}{(2R+D)^{2}}\right)\right\}$ (2)

Here, *T* = 293 K, *k*_B_ is the Boltzmann constant, *W*_0_ depends on the hydration state of the surface, *D* is the distance between the particle and the surface, $\lambda$λ is the decay length, *A* is the Hamaker constant, and *R* is the average radius of the gold nanoparticles, which is 1.28 nm. To derive the complete interaction potential function, statistical analysis of the interparticle distance distribution extracted from sequential temporal frames during the coalescence process across twenty nanoparticle groups (Fig. S5). Employing the Boltzmann distribution, the corresponding unknown parameters were extracted from the interparticle distance data: *W*_0_ = (11.71 ± 1.71) × 10^-19^ J, *λ* = 0.126 nm, and *A* = (4.73 ± 0.71) × 10^-19^ J. Combining Equations (1) and (2), the expression for the interaction potential *U*(*D*) can be derived, which is given as Equation (3) [10, 13]:

$U\text{(}D\text{) = }W_{0}e^{-\frac{D}{\lambda}}+\left\{-\frac{A}{6}\left(\frac{2R^{2}}{(4R+D)D}+\frac{2R^{2}}{(2R+D)^{2}}+\ln\frac{(4R+D)D}{(2R+D)^{2}}\right)\right\}$ (3)

Using the known parameters, the interaction potential energy *U*(*D*) between particle pairs and the corresponding force *F*(*D*) (where $F(D)=-\text{d}U(D)/\text{d }D$) were calculated using equation (3), and the results are plotted in Fig. 1(g).

**Supplementary Note 3:** **Molecular Dynamics** **(MD) Calculations**

Given that solvent environmental differences would introduce uncontrollable variables (stability of hydration layers, physicochemical properties of solvents), this study performed molecular dynamics simulations of the fusion process between two truncated-octahedral gold nanoparticles (both with 2.0 nm diameter, each containing 260 atoms) under vacuum conditions. First, in model 1, these nanoparticles were initially placed 0.3 nm apart (closest atom centers) in a $60\times60\times60$ (X×Y×Z) Å^3^ cuboid box, with their (111) planes at a 60° angle. Model 2 similarly used a $60\times60\times60$ (X×Y×Z) Å^3^ box, but with nanoparticles initially 0.5 nm apart and their (111) planes parallel. Both simulations were conducted at 300 K in the NVT ensemble, regulated by a Nosé-Hoover thermostat [14]. The interactions between Au atoms were described by the embedded atom method (EAM) potential developed by Foiles [15]. All molecular dynamics simulations were executed using the open source parallel code LAMMPS software [16]. The time step is 1 ps, and the total simulation duration is 10 ns.

To quantitatively analyze the energy conversion during the two simulation processes, a second simulation was conducted on the fusion behavior of two truncated octahedral gold nanoparticles, with the particle construction parameters maintained consistent with those used in the initial simulation. In model 1, these nanoparticles were initially placed 0.3 nm apart (closest atom centers) in a $60\times60\times60$ (X×Y×Z) Å^3^ cuboid box, with their (111) planes at a 60° angle. Model 2 similarly used a $60\times60\times60$ (X×Y×Z) Å^3^ box, but with nanoparticles initially 0.5 nm apart and their (111) planes parallel. Both simulations were conducted in the NVE ensemble with an initial temperature of 300 K. The temperature was not controlled, allowing it to evolve freely with the conversion of kinetic and potential energy. The interactions between Au atoms were described by the EAM potential developed by Foiles. All molecular dynamics simulations were executed using the open source parallel code LAMMPS software.

To determine whether there is a size limitation for the coalescence mechanism of the particles shown in Fig. 1, we initiated fusion simulations of two truncated octahedral gold nanoparticles, each with a diameter of 5.9 nm (containing 4957 atoms). These two nanoparticles were initially placed 0.3 nm apart (closest atom centers) in a $200\times200\times200$ (X×Y×Z) Å^3^ cuboid box, with a relative misorientation of 30° between their (111) planes. Simulations were conducted at 900 K in the NVT ensemble, regulated by a Nosé-Hoover thermostat. The interactions between Au atoms were described using the EAM potential developed by Foiles. All molecular dynamics simulations were executed using the open source parallel code LAMMPS. Structural analysis based on Common Neighbor Analysis (CNA) [17], and visualization were carried out using OVITO [18].

This study also conducted fusion simulations of gold nanoparticles within an ion-containing environment to elucidate the role of trace residual Br^−^. Each of the two truncated octahedral gold nanoparticles has a diameter of 2.0 nm and consists of 260 atoms. In model 1, these nanoparticles were initially placed 0.7 nm apart (closest atom centers) in a $60\times60\times60$ (X×Y×Z) Å^3^ cuboid box, with their (111) planes at a 60° angle. Model 2 similarly used a$60\times60\times60$ (X×Y×Z) Å^3^ box, but with nanoparticles initially 0.9 nm apart and their (111) planes parallel. In both models, two hydronium (H_3_O^+^) and two bromide (Br^−^) ions were introduced into the close-contact interface between the two Au nanoparticles. The H_3_O^+^ ions were constrained as rigid bodies throughout the simulations. Both simulations were conducted at 300 K in the NVT ensemble, regulated by a Nosé-Hoover thermostat. The atomic interactions were described using a combination of force fields: interactions between the Au atoms were described by the embedded-atom method (EAM) potential developed by Foiles et al., while the Madrid-2019 force field was employed for the H_3_O^+^ and Br^−^ ions [19, 20]. The cross-interactions between the Au nanoparticles and the ionic components were modeled using Lennard-Jones (LJ) and Coulombic potentials, utilizing parameters derived from the CVFF-interface force field [21] and calculated via arithmetic mixing rules. All molecular dynamics simulations were executed using the open-source parallel code LAMMPS.

The results show that both configurations achieve complete coalescence. Br^−^ is not the determining factor for fusion but mainly affects atomic rearrangement and neck growth after particle contact. After introducing Br^−^, the potential energy release during the coalescence of model 1 is 1.21 eV, and that of model 2 is 1.01 eV. For both model 1 and model 2 with Br^−^, the energy release during the coalescence exceeds that without Br^−^, and this additional energy promotes complete coalescence. The gap in energy release between the two models narrows from 0.53 eV (without Br^−^) to 0.20 eV (with Br^−^), suggesting that Br^−^ slightly promotes coalescence kinetics in the low interfacial energy system (model 2).

**Supplementary Note 4: Relationship Between Electron Flux Density and Dose Rate**

Incident electron flux density refers to the number of incident electrons per unit area per unit time. Dose rate denotes the power of energy transferred from incident electrons to the liquid cell per unit mass via inelastic scattering, typically expressed in Gy/s. In liquid-cell transmission electron microscopy, assuming that the penetration path length of primary electrons is equal to the liquid layer thickness, the relationship between electron beam dose rate and electron flux density is given by [22]:

 (4)

Here, *Ψ* is the dose rate, *φ* is the electron flux density, *e* is the elementary charge, *S* is the mass stopping power, *z_l_* is the liquid layer thickness, and *λ*_IMFP_ is the inelastic mean free path (IMFP) of electrons in the liquid. In our experiment, a 300 kV accelerating voltage was used to irradiate highly diluted aqueous solutions. Under this condition, *S* = 2.36 MeV·cm^2^·g^−1^ and *λ*_IMFP_ = 380 nm [23]. The electron flux density range for observing particle fusion and growth is 1000–1600 e^−^·Å^−2^·s^−1^. Assuming the liquid layer thickness of the liquid cell used in this experiment is 100 nm, the electron beam dose rate in the experiment is 4.8 × 10^9^–7.6 × 10^9^ Gy/s.

**Section 3**

**Figure S1****.** Transmission Electron Microscope (TEM) image of a gold nanorod showing its morphology.

**Figure S2.** The growth of Au nanoparticles through monomer attachment (particle 1) and nanoparticle coalescence (particles 2 and 3).

**Figure S3.** The schematic diagram of the time sequence atomic arrangement of nanoparticle A and nanoparticle B.

**Figure S4.** Dynamics of nanoparticle coalescence with jumping behavior. (a) The TEM image time series of the 10 pairs of particles undergoing jump-to-coalescence. (b) The variation of interparticle distance with time for the 10 pairs of particles during the approaching process, which are represented by blue dashed lines. Red solid lines indicate the change in average distance, and blue shaded areas represent the standard deviation.

**Figure S5.** Statistical distribution of interparticle separation distances (*D*) sampled from multiple sequential frames across 20 distinct particle fusion events.

**Figure S6.** The variation of the angle of the (111) crystal plane relative to the horizontal line over time for nanoparticle E and nanoparticle F.

**Figure S7.** Morphological changes of the merged particle. (a) Schematic defining particle length, neck diameter, and width (representative particle at 4.8 s). (b) Temporal evolution of particle length, neck diameter, and width during coalescence.

**Figure S8****.** Coalescence processes and interaction potential energy changes of two initial configurations of 2 nm gold nanoparticles under NVT ensemble. (a) Coalescence process of Model 1 (misoriented (111) crystallographic planes). (b) Coalescence process of Model 2 (aligned (111) crystallographic planes). (c) Time evolution of interaction potential energy between particles in Model 1. (d) Time evolution of interaction potential energy between particles in Model 2. Simulation parameters: NVT ensemble, total simulation time 10 ns (10000 steps with 1 ps time step).

**Figure S9****.** Potential and kinetic energy evolution of the two initial configurations of the 2 nm gold nanoparticles in the NVE ensemble. (a, b) Time evolution of potential and kinetic energy in Model 1 (misoriented (111) crystallographic planes). (c, d) Time evolution of potential and kinetic energy in Model 2 (aligned (111) crystallographic planes). Simulation parameters: NVE ensemble, total duration 10 ns (10000 steps with 1 ps time step).

**Figure S10.** Coalescence behavior and interparticle potential energy evolution of 2 nm Au nanoparticles with two initial configurations containing hydronium H_3_O^+^ and bromide Br^−^ ions in the NVT ensemble. (a) Coalescence process of model 1, with misoriented (111) crystallographic planes. (b) Coalescence process of model 2, with aligned (111) crystallographic planes. (c) Time evolution of the potential energy for model 1 and model 2. Simulation parameters: NVT ensemble, total simulation time 7 ns (7000 steps with 1 ps time step).

**Figure S11.** The simulation process of the coalescence of the two gold nanoparticles with a diameter of 5.9 nm at 900 K. Atoms are colored according to the common neighbor analysis (CNA), where red represents HCP structures (stacking faults and twin boundaries) and yellow represents other Au atoms. Simulation parameters: NVT ensemble, total duration 30 ns (30000 steps with 1 ps time step).

**Figure S12.** Variation in the projected area of domain Ⅰ and domain Ⅱ over time within twinned nanoparticle M.

**References**

[1] Grogan J M, Schneider N M, Ross F M and Bau H H, “Bubble and Pattern Formation in Liquid Induced by an Electron Beam,” *Nano Letters* 14 (2014): 359–364.

[2] Buxton G V and Sellers R M, “The Radiation Chemistry of Metal Ions in Aqueous Solution,” *Coordination Chemistry Reviews* 22 (1977): 195–274.

[3] Spotheim-Maurizot M, Mostafavi M, Douki T and Belloni J, “Radiation Chemistry : From Basics to Applications in Material and Life Sciences,” (2008).

[4] Fritsch B, Lee S, Körner A, Schneider N M, Ross F M and Hutzler A, “The Influence of Ionizing Radiation on Quantification for In Situ and Operando Liquid-phase Electron Microscopy,” *Advanced Materials* 37 (2025): 2415728.

[5] Hohenstein M, Seeger A and Sigle W, “The Anisotropy and Temperature Dependence of the Threshold for Radiation Damage in Gold–comparison with other FCC Metals,” *Journal of Nuclear Materials* 169 (1989): 33–46.

[6] Bauer W, Anderman A I and Sosin A, “Atomic Displacement Process in Gold,” *Physical Review* 185 (1969): 870–875.

[7] Loh N D, Sen S, Bosman M, Tan S F, Zhong J, Nijhuis C A, Král P, Matsudaira P and Mirsaidov U, “Multistep Nucleation of Nanocrystals in Aqueous Solution,” *Nature Chemistry* 9 (2017): 77–82.

[8] Israelachvili J, “Intermolecular and Surface Forces, 3rd Edition,” (2011).

[9] Bishop K J M, Wilmer C E, Soh S and Grzybowski B A, “Nanoscale Forces and Their Uses in Self-assembly,” *Small* 5 (2009): 1600–1630.

[10] Zhu C, Liang S, Song E, Zhou Y, Wang W, Shan F, Shi Y, Hao C, Yin K, Zhang T, Liu J, Zheng H and Sun L, “In-situ Liquid Cell Transmission Electron Microscopy Investigation on Oriented Attachment of Gold Nanoparticles,” *Nature Communications* 9 (2018): 421.

[11] Derjaguin B V, Churaev N V and Muller V M, “The Derjaguin–Landau–Verwey–Overbeek (DLVO) Theory of Stability of Lyophobic Colloids,” *Surface Forces* Boston, MA: Springer US (1987): 293–310.

[12] Anand U, Lu J, Loh D, Aabdin Z and Mirsaidov U, “Hydration Layer-mediated Pairwise Interaction of Nanoparticles,” *Nano Letters* 16 (2016): 786–790.

[13] Mondain-Monval O, Espert A, Omarjee P, Bibette J, Leal-Calderon F, Philip J and Joanny J F, “Polymer-induced Repulsive Forces: Exponential Scaling,” *Physical Review Letters* 80 (1998): 1778–1781.

[14] Nosé S, “Constant Temperature Molecular Dynamics Methods,” *Progress of Theoretical Physics Supplement* 103 (1991): 1–46.

[15] Foiles S M, Baskes M I and Daw M S, “Embedded-atom-method Functions for the Fcc Metals Cu, Ag, Au, Ni, Pd, Pt, and Their Alloys,” *Physical Review B* 33 (1986): 7983–7991.

[16] Plimpton S, “Fast Parallel Algorithms for Short-range Molecular Dynamics,” *Journal of Computational Physics* 117 (1995): 1–19.

[17] Honeycutt J D and Andersen H C, “Molecular Dynamics Study of Melting and Freezing of Small Lennard-Jones Clusters,” *The Journal of Physical Chemistry*, 91 (1987): 4950–4963.

[18] Stukowski A, “Visualization and Analysis of Atomistic Simulation Data with OVITO–the Open Visualization Tool,” *Modelling and Simulation in Materials Science and Engineering* 18 (2010): 015012.

[19] Blazquez S, de Lucas M, Vega C and Gámez F, “Acidifying the Madrid-2019 Force Field: A Rigid Model for H_3_O^+^ with Scaled Charges,” *The Journal of Chemical Physics* 162 (2025): 171101.

[20] Blazquez S, Conde M M, Abascal J L F and Vega C, “The Madrid-2019 Force Field for Electrolytes in Water Using TIP4P/2005 and Scaled Charges: Extension to the Ions F^−^, Br^−^, I^−^, Rb^+^, and Cs^+^,” *The Journal of Chemical Physics* 156 (2022): 044505. [21] Heinz H, Lin T J, Kishore Mishra R and Emami F S, “Thermodynamically Consistent Force Fields for the Assembly of Inorganic, Organic, and Biological Nanostructures: The INTERFACE Force Field,” *Langmuir* 29 (2013): 1754–1765.

[22] Fritsch B, Hutzler A, Wu M, Khadivianazar S, Vogl L, Jank M P M, März M and Spiecker E, “Accessing Local Electron-beam Induced Temperature Changes during In Situ Liquid-phase Transmission Electron Microscopy,” *Nanoscale Advances* 3 (2021): 2466–2474.

[23] Yesibolati M N, Laganá S, Kadkhodazadeh S, Mikkelsen E K, Sun H, Kasama T, Hansen O, Zaluzec N J and Mølhave K, “Electron Inelastic Mean Free Path in Water,” *Nanoscale* 12 (2020): 20649–20657.
